# Spatial and temporal distribution of falciparum malaria in China

**DOI:** 10.1186/1475-2875-8-130

**Published:** 2009-06-12

**Authors:** Hualiang Lin, Liang Lu, Linwei Tian, Shuisen Zhou, Haixia Wu, Yan Bi, Suzanne C Ho, Qiyong Liu

**Affiliations:** 1 National Institute for Communicable Disease Control and Prevention, China CDC; State Key Laboratory for Infectious Disease Prevention and Control, Beijing, PR China; 2 Stanley Ho Centre for Emerging Infectious Diseases, School of Public Health, Chinese University of Hong Kong, Hong Kong SAR, PR China; 3 National Malaria Office, National Institute for Parasitic Diseases, Shanghai, PR China; 4 Yunnan Center for Disease Control and Prevention, Kunming, PR China

## Abstract

**Background:**

Falciparum malaria is the most deadly among the four main types of human malaria. Although great success has been achieved since the launch of the National Malaria Control Programme in 1955, malaria remains a serious public health problem in China. This paper aimed to analyse the geographic distribution, demographic patterns and time trends of falciparum malaria in China.

**Methods:**

The annual numbers of falciparum malaria cases during 1992–2003 and the individual case reports of each clinical falciparum malaria during 2004–2005 were extracted from communicable disease information systems in China Center for Diseases Control and Prevention. The annual number of cases and the annual incidence were mapped by matching them to corresponding province- and county-level administrative units in a geographic information system. The distribution of falciparum malaria by age, gender and origin of infection was analysed. Time-series analysis was conducted to investigate the relationship between the falciparum malaria in the endemic provinces and the imported falciparum malaria in non-endemic provinces.

**Results:**

Falciparum malaria was endemic in two provinces of China during 2004–05. Imported malaria was reported in 26 non-endemic provinces. Annual incidence of falciparum malaria was mapped at county level in the two endemic provinces of China: Yunnan and Hainan. The sex ratio (male vs. female) for the number of cases in Yunnan was 1.6 in the children of 0–15 years and it reached 5.7 in the adults over 15 years of age. The number of malaria cases in Yunnan was positively correlated with the imported malaria of concurrent months in the non-endemic provinces.

**Conclusion:**

The endemic area of falciparum malaria in China has remained restricted to two provinces, Yunnan and Hainan. Stable transmission occurs in the bordering region of Yunnan and the hilly-forested south of Hainan. The age and gender distribution in the endemic area is characterized by the predominance of adult men cases. Imported falciparum malaria in the non-endemic area of China, affected mainly by the malaria transmission in Yunnan, has increased both spatially and temporally. Specific intervention measures targeted at the mobile population groups are warranted.

## Background

Malaria incidence appears to be decreasing worldwide as a result of mass interventions and other factors [1,2]. The decreasing pattern of incidence is encouraging and the international community has recently been challenged to re-evaluate the prospects for malaria eradication [3]. Falciparum malaria is the most deadly among the four main types of human malaria. The number of clinical events caused by *Plasmodium falciparum *was estimated to be 515 million worldwide in 2002 [4]. Falciparum malaria transmission is a global problem requiring a global intervention strategy with regional targets. While most falciparum attacks are concentrated in the African region (70%), the densely populated Southeast Asia region contributed 25% of the clinical attacks in 2002 [4]. The Southeast Asia region has also been the focus for the origin of drug-resistant malaria, which may have contributed to the rising mortality from malaria in the African region since 1990 [5]. The epidemic situation in China is largely affected by that in the nearby Southeast Asian countries [6].

Although great success has been achieved since the launch of National Malaria Control Programme in 1955, malaria remains a serious public health problem in China [7-9]. Falciparum malaria, the most deadly among the four main types of human malaria, accounted for 14.9% of all blood-test confirmed malaria cases in 1998 [10]. Falciparum malaria had been endemic in fifteen provinces of China in the early 1950s. Integrated measures for malaria control, involving the vector as well as malaria infections, were effective in eliminating falciparum malaria in central China [11]. The endemic area of falciparum malaria was restricted to eight provinces by 1980, and only to two provinces, Yunnan and Hainan, by 1998 [10]. Imported falciparum malaria, however, has spread to more provinces in the non-endemic area due to the increasing population movement. Reported in only two provinces in 1984, imported falciparum malaria was found in 16 provinces in 1998 [10]. A changing malaria landscape requires an updated spatial and temporal description for effective resource allocation and intervention. This paper aims to analyse the geographic distribution, demographic patterns and time trends (1992–2005) of falciparum malaria in China.

## Methods

The monthly numbers of falciparum malaria cases from 1992 to 2003 were obtained from the Statistics on the Notifiable Communicable Disease Surveillance released by the China Center for Diseases Control and Prevention (China CDC). Individual case reports of each clinical falciparum malaria from 2004 to 2005 were obtained from the Reporting System of Communicable Diseases and Unexpected Public Health Events established by the China CDC in 2004. This information and surveillance system requests that certain communicable diseases, including malaria, are reported online. The coverage of the system has reached county level in general and township level in some endemic region of malaria. Extracted information for each case includes age, gender and the places where the infection was diagnosed and acquired, respectively. The age and gender distribution of falciparum malaria in the endemic areas were analysed.

Autochthonous malaria was defined as any case infected inside the province in which it was diagnosed; in contrast, imported malaria was defined as a malaria case whose origin could be traced to an area of transmission outside the province in which the diagnosis of malaria was made [12,13]. The reported cases of confirmed malaria per 1,000 resident population were computed for each year by administrative levels (province and county) and averaged over the reporting years. The 1992–2003 data had no detailed spatial information at province or county level, so only the 2004–2005 data were used for mapping. The number of *P. falciparum*-specific malaria cases was mapped at province level to show the whole picture first; the annual parasite incidence (*Pf*API), expressed as the number infected per 1,000 people per annum (pa) [12,14], was then mapped at county level in the two endemic provinces, Yunnan and Hainan. PfAPI was classified into three categories: no autochthonous falciparum cases reported, < 0.1 autochthonous falciparum cases per 1,000 people pa, and ≥ 0.1 autochthonous falciparum cases per 1,000 people pa. These three categories were defined as stable, non-stable, or no risk of falciparum malaria transmission [15]. The number of confirmed cases and *Pf*API, respectively, were mapped by matching them to their corresponding province- and county-level administrative units in a geographic information system (ArcGIS 9.0, ESRI, Redlands, CA).

Time-series analysis was conducted to investigate the relationship between the falciparum malaria in the endemic provinces and the imported malaria in non-endemic provinces of China. An auto-regressive integrated moving average (ARIMA) model was fit first to the predictor variable. The model was then applied to the dependent variable before the two series were cross-correlated to determine whether an association exists. ARIMA was designed to deal with highly seasonal data. Modeling with ARIMA involves the estimation of a series of parameters to account for the inherent dynamics in the time series, including the trends and autoregressive and moving average processes. The general model introduced by Box and Jenkin [16] includes autoregressive and moving average parameters, and explicitly includes differencing in the formulation of the model. An ARIMA (*p, d, q*) model comprises three types of parameters: the autoregressive parameters (*p*), number of differencing passes (*d*), and moving average parameters (*q*). The multiplicative seasonal ARIMA (*p, d, q*)(*P, D, Q*)_*s *_model is an extension of the ARIMA method to time series in which a pattern repeats seasonally over time. Analogous to the simple ARIMA parameters, the seasonal parameters are: seasonal autoregressive (*P*), seasonal differencing (*D*), and seasonal moving average parameters (*Q*). The length of the seasonal period is represented by *s*. The input series of incidence rates in the two malaria endemic provinces, at lags of 0–2 months, were fitted in the ARIMA model of the incidence rate in the non-endemic provinces of China. The selection of ARIMA processes was conducted using Akaike's information criterion (AIC), which measures how well the model fits the series.

## Results

**Figure 1:**
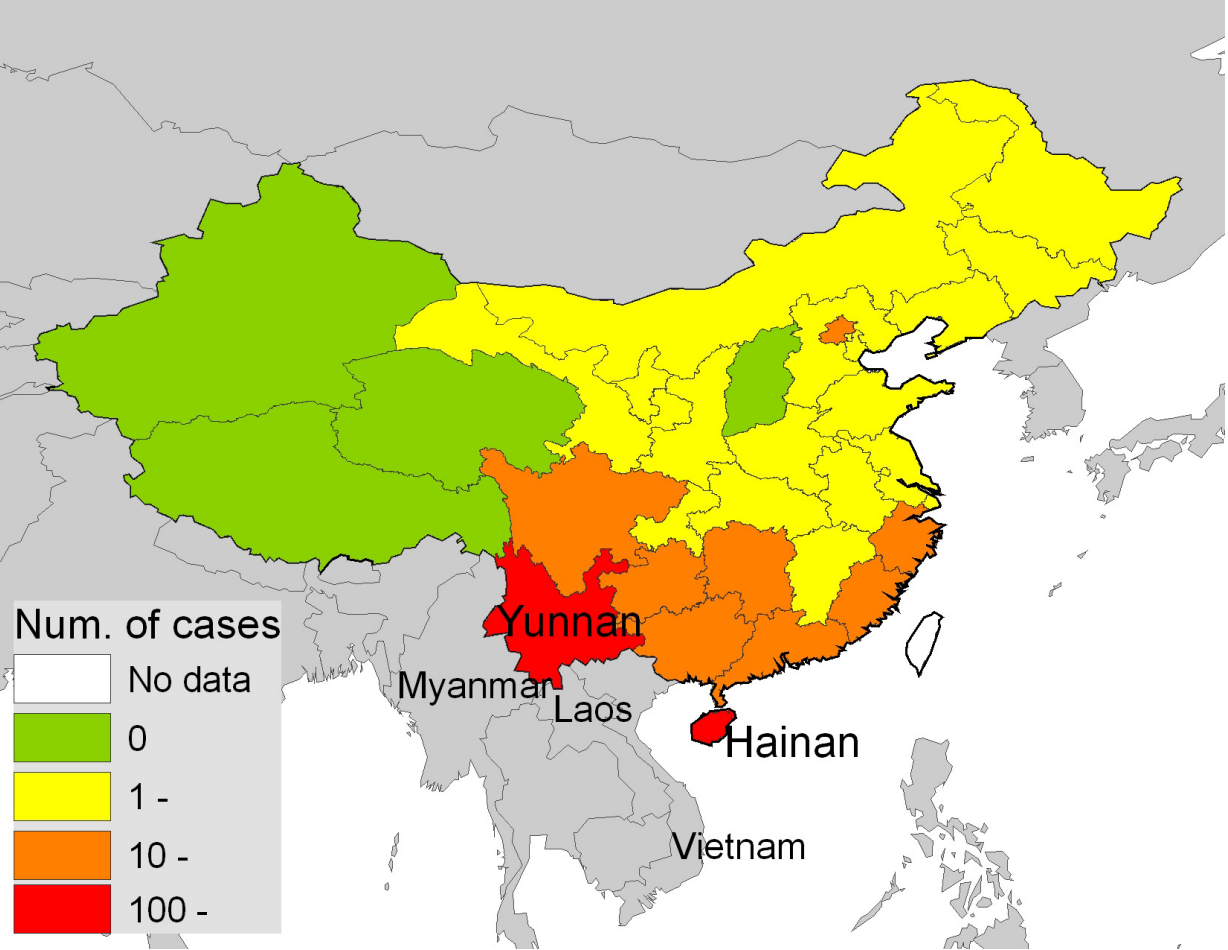
**Annual average number of falciparum malaria cases in China from during 2004–05**. All the falciparum cases were imported except in the endemic provinces of Yunnan and Hainan.

Figure 1 shows the province-level distribution of falciparum malaria cases in China during 2004–05. Falciparum malaria cases were reported all over China except in four provinces in the west (Xinjiang, Xizang, Qinghai and Shanxi). All provinces in northern China, except the Beijing municipal area, reported less than 10 falciparum cases annually. In the seven southern provinces close to the endemic areas of Yunnan and Hainan, 10–100 falciparum malaria cases were reported annually. All the cases were imported except in the endemic provinces of Yunnan and Hainan, for which the total numbers of imported and autochthonous cases were presented.

**Figure 2:**
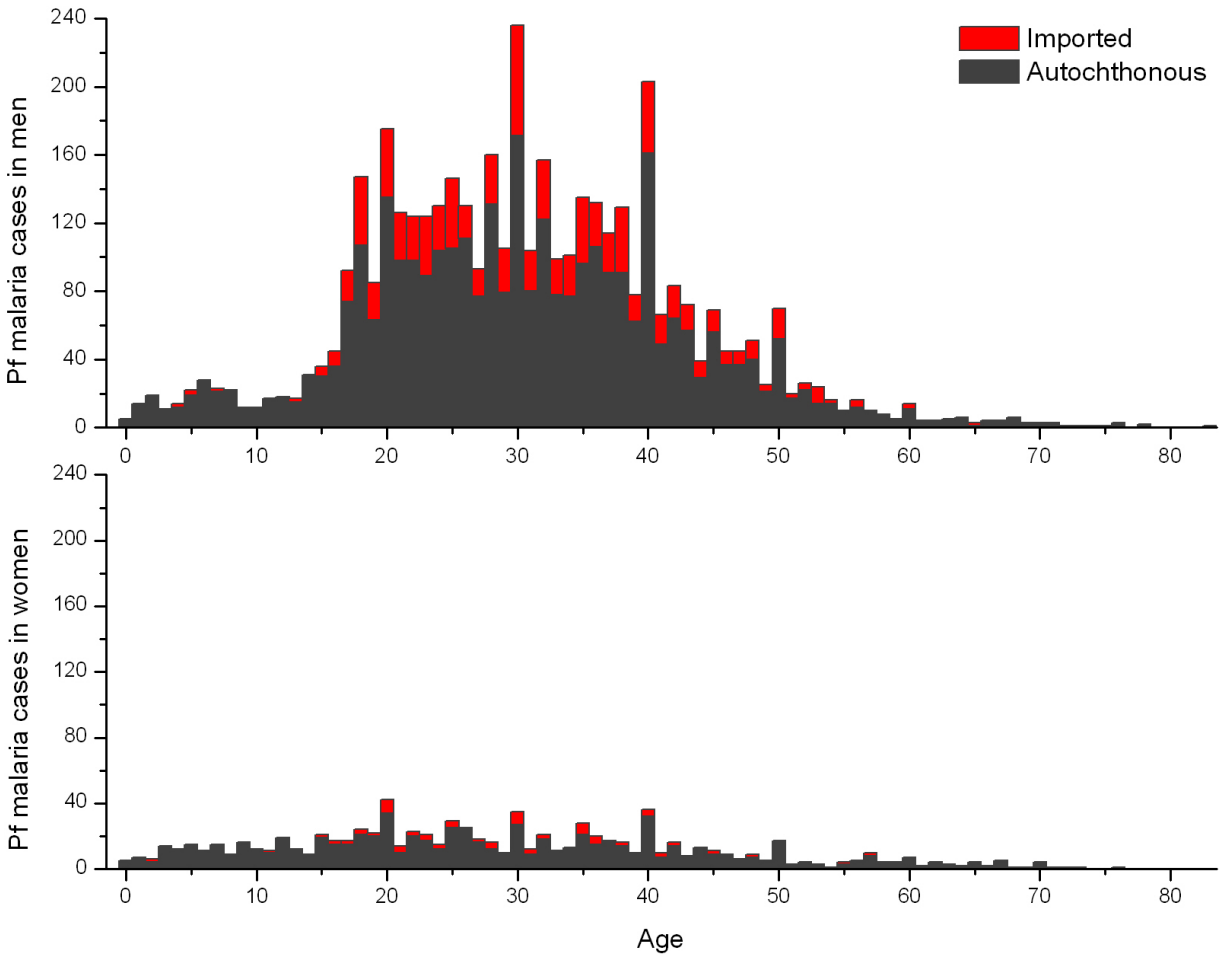
**Age and gender distribution of imported and autochthonous falciparum malaria cases in Yunnan, China**.

**Figure 3:**
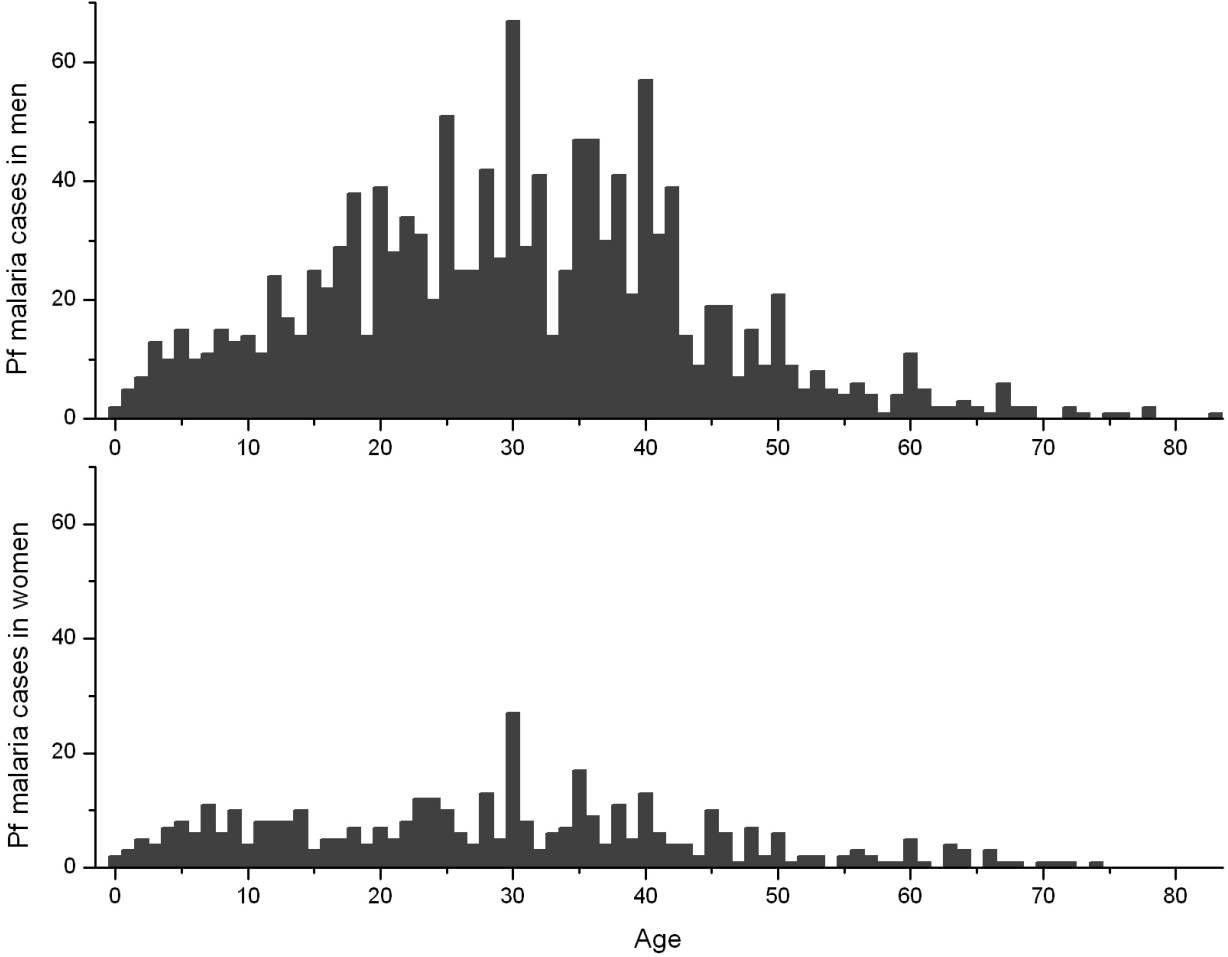
**Age and gender distribution of Pf malaria cases in Hainan, China**. No imported malaria was reported in Hainan; it was believed that all the reported cases were autochthonous.

Figure 2 shows the age and gender distribution of imported and autochthonous falciparum malaria cases in Yunnan Province. Adults over 15 years of age accounted for 93% and 78% of the total cases in men and women, respectively. Young adults of 15–50 years were predominant. The number of imported cases from abroad was negligible in the children of 0–15 years; in the adults over 15 years of age, however, imported cases accounted for a large fraction: 22% in men and 13% in women. Myanmar was the predominant location of infection for the imported cases. The total number of falciparum malaria cases was substantially higher in men than in women. The sex ratio (male vs. female) was 1.6 in the children of 0–15 years and it reached 5.7 in the adults over 15 years of age. Figure 3 shows the age and gender distribution of falciparum malaria cases in Hainan Province. No imported malaria was reported in Hainan; it was believed that all the reported cases were autochthonous. The predominance of adult cases of falciparum malaria was similar to that in Yunnan: adults over 15 years of age accounted for 84% and 74% of the total cases in men and women, respectively, in Hainan. The number of cases was also substantially higher in men than in women. The sex ratio (male vs. female) was 2.0 in the children of 0–15 years and it reached 3.8 in the adults over 15 years of age.

**Figure 4:**
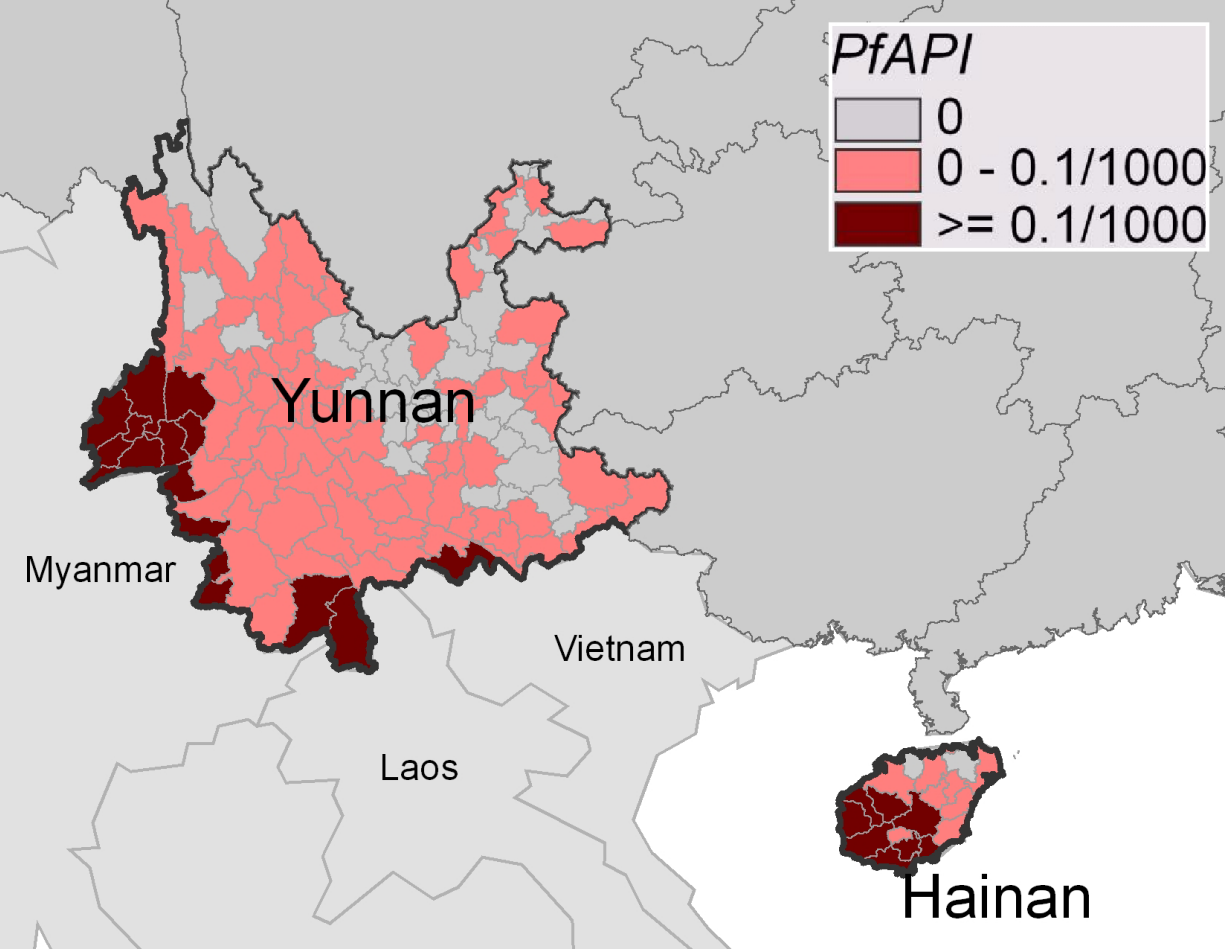
**County-level distribution of PfAPI, computed by autochthonous falciparum cases only, in Yunnan and Hainan Provinces during 2004–05**. Counties were classified into three categories according to the falciparum malaria risk: stable (dark-red areas, where PfAPI ≥ 0.1 per thousand pa), unstable (pink areas, where PfAPI < 0.1 per thousand pa), or no risk (light grey).

Figure 4 shows the county-level distribution of PfAPI, computed by autochthonous falciparum cases only, in Yunnan and Hainan Provinces during 2004–05. Counties were classified into three categories according to the falciparum malaria risk: stable (dark-red areas, where *Pf*API ≥ 0.1 per thousand pa), unstable (pink areas, where *Pf*API < 0.1 per thousand pa), or no risk (light grey). The classification and choropleth were consistent with those in the global map of falciparum malaria by Guerra [15]. Falciparum malaria was not endemic in all the counties in Yunnan and Hainan as many counties reported no autochthonous cases. Counties of stable falciparum malaria transmission cluster in the bordering region of Yunnan and the hilly-forested south of Hainan.

**Figure 5:**
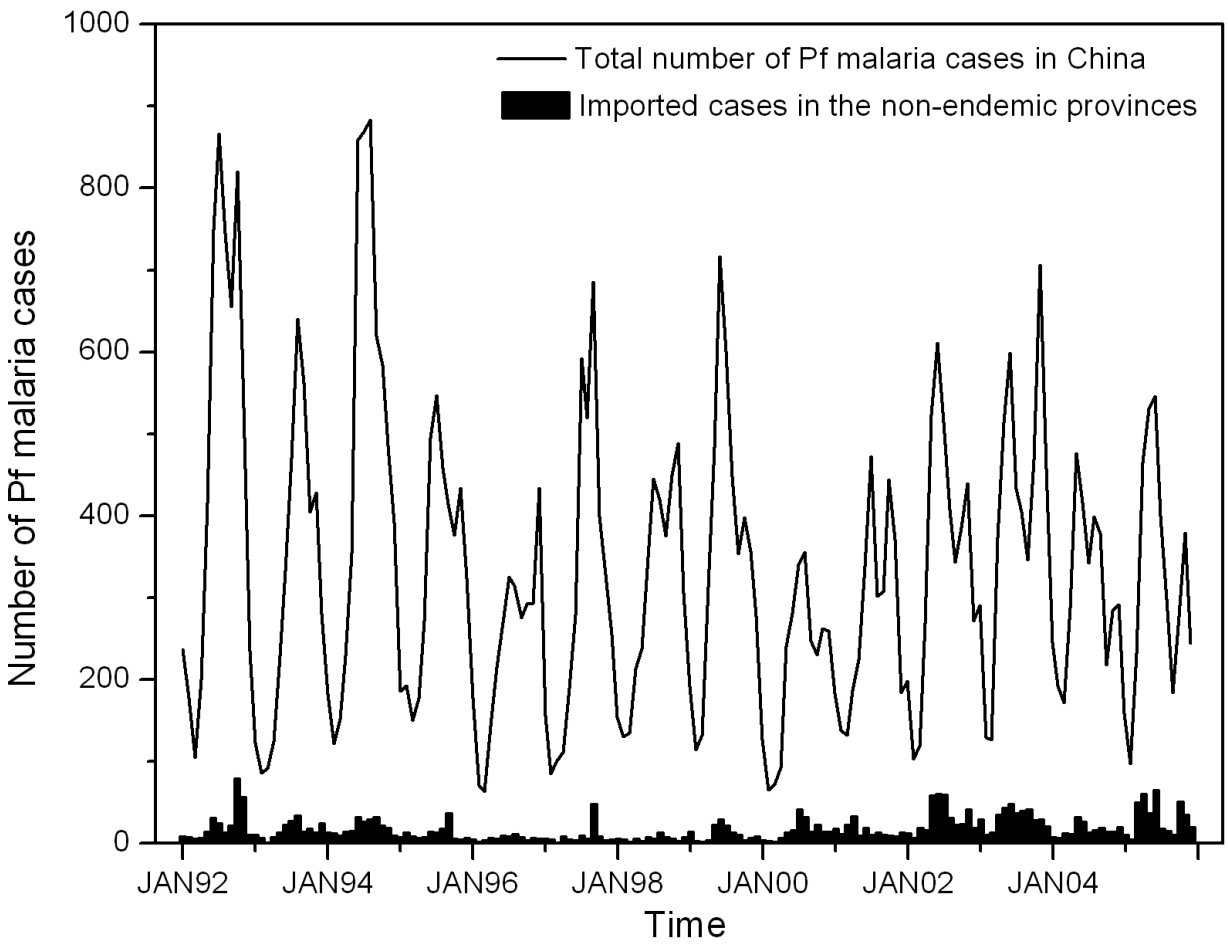
**Monthly time trends of the total number of falciparum malaria cases in China and the number of imported cases in the non-endemic provinces of China from 1992 to 2005**.

Figure 5 portrays the monthly time trends of the total number of falciparum *malaria cases in China and the number of imported cases in the non-endemic provinces of China from 1992 to 2005. While the total number of *falciparum *malaria cases remained relatively stati*c (P = 0.20 for linear trend), the number of imported cases in the non-endemic areas appeared to be increasing (P < 0.01 for linear trend). The fraction of imported cases increased from 5% in 1992 to 8% in 2005 (P < 0.01 for linear trend). Time-series analysis was conducted to investigate the predominant source region of imported malaria. Table 1 lists two ARIMA models where the incidence rates in the two malaria endemic provinces, Yunnan and Hainan, are included as input series, respectively, to predict the imported malaria incidence in the non-endemic provinces of China. Of all the models tested, the seasonal ARIMA *(1,1,1)(0,1,1)*_12 _model for malaria incidence fits the data best according to AIC and goodness-of-fit criteria. Model II fits the data better than Model I because of the lower AIC value. The local moving average parameter is 0.843, the seasonal moving average is 0.682, and the autoregression is 0.207, all of which are statistically significant. As seen in Model I, the Hainan series is not associated with the imported malaria in the non-endemic provinces of China (*p *= 0.218); while in Model II, the Yunnan series is positively associated with the imported malaria of concurrent months in the non-endemic provinces of China (β = 0.459, *p *= 0.001).

**Table 1:** ARIMA regression of the imported malaria in the non-endemic provinces on the number of falciparum malaria in the endemic Yunnan and Hainan Provinces (1992–2005)

	Model I			Model II		
		
		S.E.	*p*		S.E.	*p*
Moving average	0.864	0.054	<.0001	0.843	0.057	<.0001
Seasonal moving average	0.651	0.068	<.0001	0.682	0.064	<.0001
Auto-regression	0.273	0.102	0.008	0.207	0.103	0.046
Hainan (lag 0)	0.148	0.120	0.218			
Yunnan (lag 0)				0.459	0.141	0.001
AIC		57.7			49.6	

## Discussion

The endemic area of falciparum malaria in China has remained restricted to two provinces, Yunnan and Hainan, since 1998. Counties of stable malaria transmission clustered in the bordering region of Yunnan and the hilly-forested areas in the south of Hainan. While frequent cross-border population movement exacerbates the malaria problem in Yunnan, the island province of Hainan remains relatively isolated, which makes falciparum malaria elimination on this island an achievable goal in the near future [17]. Efficient allocation of health resources for malaria control using appropriate combinations of interventions requires accurate information on the geographic distribution and intensity of malaria risk [4,18,19]. The county-level local map of falciparum malaria risk in the two endemic provinces of China complements the global map of Guerra [15] by using the same classification and choropleth.

The age distribution suggests the predominance of young adult cases in both endemic provinces of China. This pattern is particularly evident in men but not in women. Part of this pattern may be attributed to a large number of non-immune adults such as travelers and laborers moving from low-transmission to high-transmission areas. While population movement from and to the relatively isolated Hainan island may be minor, the movement between counties within this island province could partially explain the predominance of young adult cases.

The gender distribution of malaria cases was striking in the endemic areas of China. Among the adults over 15 years of age, the sex ratio (male vs. female) reached 3.8 in Hainan and 5.7 in Yunnan. It is possible that underreporting differentiates by gender due to varied access to health care services [20,21]. Division of labour, leisure patterns, and sleeping arrangements may lead to different patterns of exposure to mosquitoes for men and women [22]. Compared with women in some societies, men have a greater occupational risk if they work in mines, fields or forests at peak biting times, or migrate to areas of high endemicity for work [23]. For example, the exophilic *Anopheles dirus *is the principal vector for falciparum malaria in the hilly-forested endemic area of Hainan and SE Asia [24]; more working hours outdoors may have contributed to the higher malaria risk among men. The sex ratio of falciparum malaria numbers in the children of 0–15 years was not as skewed as that in adults; the sex ratio of incidence rate was unknown because of the lack of age-specific population data.

Imported falciparum malaria has spread to more provinces in the non-endemic area in China. Reported in only two non-endemic provinces in 1984, imported falciparum malaria was found in 16 provinces in 1998 [10]. As revealed in the present paper, imported *Pf *malaria cases were reported in 26 provinces of China during 2004–05. In line with the spatial expansion, there was also a temporal increase of the number of imported malaria cases in China. The fraction of imported cases increased from 5% in 1992 to 8% in 2005. Increased population movement, domestic and international, may have contributed to the spread of imported falciparum *malaria in China *[25,26]. *The origin of infection was traced to the endemic areas of Yunnan and Hainan Provinces of China, SE Asia and Africa *[27]. *Identifying and understanding the influence of these population movements can improve the malaria intervention measures *[28].

Time-series analysis revealed that the imported falciparum malaria in China was influenced mainly by the endemic malaria in Yunnan Province, which was in turn affected by the bordering SE Asia countries: Myanmar, Laos and Vietnam. Time-series analysis has been used extensively in the study of infectious diseases and ARIMA models are useful tools to analyse time-series data containing seasonal trends [29-31]. In the current study, falciparum *malaria in *Yunnan was positively correlated with the imported malaria of concurrent months in the non-endemic provinces of China. Imported malaria also occur between the endemic areas of Yunnan, China and SE Asia due to the frequent cross-border population movement. Imported cases accounted for a large fraction of adult infections in Yunnan: 22% in men and 13% in women. In a survey among mobile population in the border area of Yunnan, *P. falciparum *parasite rates (*Pf*PR) was 3.1% for the SE Asian entering Yunnan and 0.7% for the local people returning from SE Asia visits [32]. Intervention measures tailored to these mobile populations are warranted in the future malaria control programmes.

The primary limitation of the current study is that all analyses were based solely on the national surveillance data, which is compromized by underreporting of malaria. As high as 90% of malaria cases may go unreported in China [33]. The skewed gender distribution of falciparum malaria in the endemic areas could be partially explained by the gender-differentiated underreporting. Moreover, the spatial and demographic analyses were based on a short period of time, 2004–2005. Despite these limitations, this study provides valuable information on the geographic distribution, demographic patterns and time trends of falciparum malaria in China.

## Conclusion

The endemic area of falciparum malaria in China has remained restricted to two provinces, Yunnan and Hainan. Stable falciparum malaria transmission occurs in the bordering region of Yunnan and the hilly-forested south of Hainan. The age and gender distribution in the endemic area is characterized by the predominance of adult men cases. Imported falciparum malaria in the non-endemic area of China, affected mainly by the malaria transmission in Yunnan, has increased both spatially and temporarily. Specific intervention measures targeted at the mobile population groups are warranted.

## Competing interests

The authors declare that they have no competing interests.

## Authors' contributions

HLL, LL, LWT and QYL conceived the study, undertook statistical analysis and drafted the manuscript. SSZ, HXW, YB and SCH assisted with data collection and statistical analysis. All authors contributed to the writing of the manuscript and approved the submitted version of the manuscript.
